# Novel Use of a Minnesota Tube for Enteral Feeding in a Critically Ill Patient With Gastroesophageal Bleeding

**DOI:** 10.7759/cureus.36340

**Published:** 2023-03-18

**Authors:** Daniel Udrea, Nicholas J Montano, Megan Cochran-Yu

**Affiliations:** 1 Department of Critical Care Anesthesia/Department of Emergency Medicine, Loma Linda University Medical Center, Loma Linda, USA; 2 Department of General Surgery, Division of Trauma and Acute Care Surgery, Loma Linda University Medical Center, Loma Linda, USA

**Keywords:** massive upper gastrointestinal hemorrhage, blakemore tube, minnesota tube, liver transplantation, enteral nutrition, septic shock, balloon tamponade, hemorrhagic shock, gastric balloon

## Abstract

Balloon tamponade of bleeding varices is a temporizing measure acting as a bridge for the treatment of massive gastrointestinal (GI) hemorrhage. After treatment, utilization of a gastric tube for feeding is challenging due to the risk of variceal rebleeding during placement. No literature to date has explored the use of the suction ports of a tamponade device as an alternative form of enteral access for medication and feeding administration in critically ill patients. We report a case of the novel use of a Minnesota tube for enteral feeds and medication administration in a critically ill patient awaiting liver transplantation after massive upper GI bleeding.

## Introduction

A Minnesota tube is a medical device that can be used as a balloon tamponade device in the stomach and esophagus. It is a thin, flexible tube with a tapered tip and a balloon at the end. To use the Minnesota tube as a balloon tamponade device, it is inserted through the mouth and advanced into the esophagus. The balloon is then inflated with air or saline solution, which helps to occlude the bleeding vessel and stop the bleeding [1]. This procedure is often used to treat bleeding in the upper gastrointestinal (GI) tract, such as bleeding from gastric or esophageal varices. The Minnesota tube, with an inflated balloon, can be left in place up to 24 hours to stabilize variceal bleeding. It is then removed once the bleeding has stopped and the patient's condition has stabilized. It can be an effective way to control bleeding in the upper GI tract and is often used in cases where other methods have been unsuccessful. In this case report, we present a case of the novel use of a Minnesota tube for enteral feeds and medication administration in a critically ill patient awaiting liver transplantation after massive upper GI bleeding.

## Case presentation

A 46-year-old male with a history of primary sclerosing cholangitis, esophageal varices, and gallbladder adenocarcinoma presented with abdominal pain, distension, and upper GI bleeding. He was admitted to the ICU where his bleeding was controlled via esophagogastroduodenoscopy (EGD) with repeated variceal band ligations. He developed liver failure and spontaneous bacterial peritonitis and was started on broad-spectrum antimicrobials. He was transferred to the tertiary liver center for transplant evaluation.

**Figure 1:**
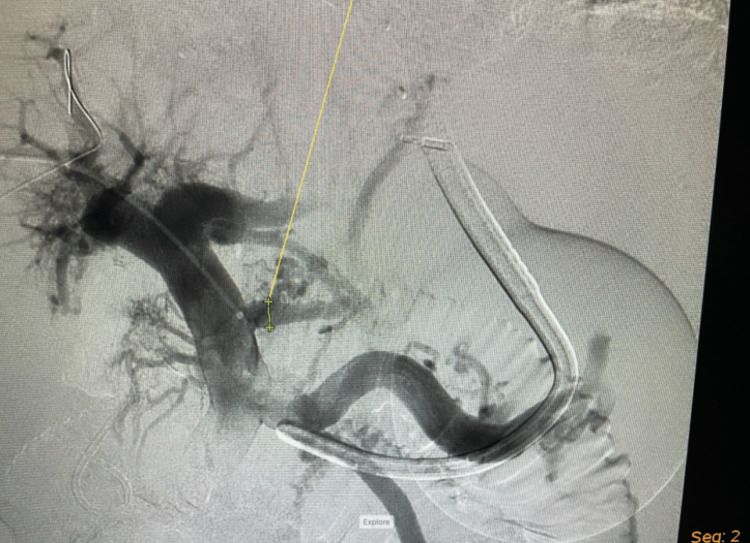
Interventional radiology angiography of patient’s portal circulation and varices with the gastric balloon of the Minnesota tube visible, with overlying gastric varices. You can visualize the appendage distal to the gastric suction port in this radiographic image.

Upon arrival, he was in septic shock. He subsequently developed hepatorenal syndrome and hepatic encephalopathy and was started on hemodialysis. He had recurrent hematemesis which was again temporized with band ligation. On day 8, he had an episode of massive hematemesis, necessitating intubation, and repeat EGD that demonstrated brisk bleeding from esophageal varices. Both variceal banding and hemostat spray were trialed and were unsuccessful. During endoscopy, the patient developed a tension pneumothorax necessitating a thoracostomy and chest tube placement. The patient had worsening hypoxemic respiratory failure with elevated ventilator peak pressures. Emergent flexible bronchoscopy was performed with no identified cause of the respiratory failure. A bedside exam revealed a tense abdomen, and an emergent resuscitative large-volume paracentesis was performed with resolution of the respiratory failure. A Minnesota tube, herein referred to as "MT" (BD, Franklin Lakes, NJ), was placed with subsequent tamponade of the bleeding and hemodynamic stability. The MT was stabilized with one kilogram of traction, and the length was marked to identify any migration. He was transported to interventional radiology where definitive control was obtained with gel foam, Avitene (BD, Franklin Lakes, NJ), and Amplatzer (Abbott, Plymouth, MN) devices (Figure 1). The MT was deflated after 24 hours.

**Figure 2:**
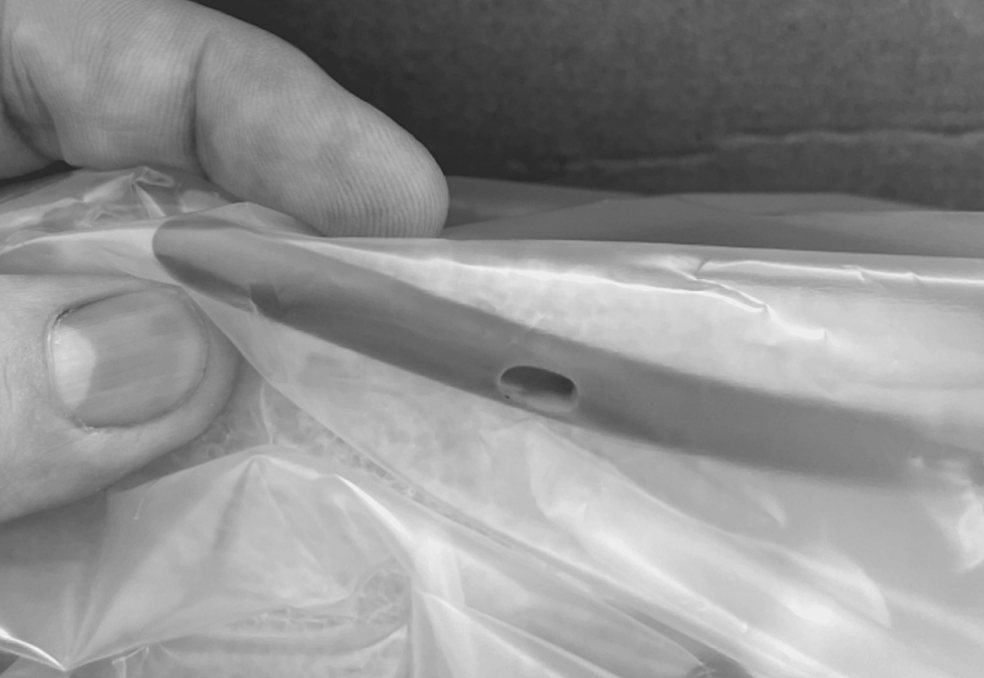
Distal suction port of the Minnesota tube with an appendage.

Decision was made to keep the MT in place and to trial use for feeding and medication administration. This was accomplished utilizing the ‘gastric suction’ port of the MT that was connected to a Kangaroo ePump^TM^ (Cardinal Health, Dublin, OH). Feeds were successfully advanced to a goal rate of 40 cc/hr. The pump did not raise an alarm, and no concerns were voiced by the nursing team as to malfunction or resistance. When medications were administered, the gastric suction port was flushed with 40cc of saline post administration to bypass the “appendix” of the tube (Figure 2). It was used successfully for eight days until the patient received his liver transplant. The patient did well post operatively and was discharged three months later fully neurologically intact and able to ambulate without assistance.

## Discussion

Massive upper GI bleeding requires immediate intervention and resuscitation to sustain life. In patients who are refractory to conventional therapies such as endoscopy, the use of balloon tamponade devices can be potentially life-saving as a bridge to treatments such as the transjugular intrahepatic portosystemic shunt procedure, gel foam or occlusion devices, and transplants. Special care should be taken with manipulation of an MT as prolonged or over-inflation can lead to migration and necrosis of surrounding tissue [2]. Each device has unique variations in the number of balloons, suction ports, and volumes of inflation. The placement of this tube is technically challenging, requiring multiple steps, and highlights the importance of simulation training [3], along with a review of the steps involved prior to attempting on a patient [4].

Its use as a device for enteral feeding and medication administration has not been previously reported in the literature. While typically a naso-gastric or oro-gastric feeding tube may be placed in patients for enteral feeding, the decision was made to leave the MT in place for two purposes. The first purpose was in case repeat tamponade was needed for recurrent massive GI bleeding. The second purpose was to be used as an enteral feeding and medication administration device. A second MT was obtained, and our nursing team was able to demonstrate and easily connect the gastric suction port to the Kangaroo enteral feeding pump [5]. A trial of feeding was performed with the second tube, and it tolerated increasing volumes with no resistance or machine alarming noted. It was noted that the “appendix” of the tube may hold onto crushed medications, causing concern that the patient may inadvertently obtain a “bolus” of medication that could potentially cause harm. We determined that 40cc of free water flushes after the administration of medication was satisfactory without leaving any medication stuck in the MT’s appendix. This demonstration using the second tube allowed the team to feel comfortable using the first tube already in the patient. Examination of the tube after it was removed during the liver transplant showed that it had remained intact, with no breakdown, clots, or medications stuck in the tube.

## Conclusions

In summary, this case report describes a novel use of the Minnesota tube as a device for enteral feeds and medication administration in a critically ill patient awaiting liver transplantation after massive upper GI bleeding. The tube was successfully used for eight days without any complications or machine alarms. This case report highlights the potential for the Minnesota tube to be used for alternative purposes and suggests further studies on its safety and efficacy as a multipurpose device. Additionally, we emphasize the importance of simulation training and careful consideration of the steps involved in placing the tube before attempting it on a patient. We hope that this report may be used for further study on safety and alternative uses of this multipurpose device.
